# Mitoquinone Protects Podocytes from Angiotensin II-Induced Mitochondrial Dysfunction and Injury via the Keap1-Nrf2 Signaling Pathway

**DOI:** 10.1155/2021/1394486

**Published:** 2021-08-13

**Authors:** Zijing Zhu, Wei Liang, Zhaowei Chen, Jijia Hu, Jun Feng, Yun Cao, Yiqiong Ma, Guohua Ding

**Affiliations:** ^1^Division of Nephrology, Renmin Hospital of Wuhan University, Wuhan 430060, China; ^2^Nephrology and Urology Research Institute of Wuhan University, Wuhan 430060, China

## Abstract

Podocyte mitochondrial dysfunction plays a critical role in the pathogenesis of chronic kidney disease (CKD). Previous studies demonstrated that excessive mitochondrial fission could lead to the overproduction of reactive oxygen species (ROS) and promote podocyte apoptosis. Therefore, the maintenance of stable mitochondrial function is a newly identified way to protect podocytes and prevent the progression of CKD. As a mitochondria-targeted antioxidant, mitoquinone (MitoQ) has been proven to be a promising agent for the prevention of mitochondrial injury in cardiovascular disease and Parkinson's disease. The present study examined the effects of MitoQ on angiotensin II- (Ang II-) induced podocyte injury both *in vivo* and *in vitro*. Podocyte mitochondria in Ang II-infused mice exhibited morphological and functional alterations. The observed mitochondrial fragmentation and ROS production were alleviated with MitoQ treatment. *In vitro*, alterations in mitochondrial morphology and function in Ang II-stimulated podocytes, including mitochondrial membrane potential reduction, ROS overproduction, and adenosine triphosphate (ATP) deficiency, were significantly reversed by MitoQ. Moreover, MitoQ rescued the expression and translocation of Nrf2 (nuclear factor E2-related factor 2) and decreased the expression of Keap1 (Kelch-like ECH-associated protein 1) in Ang II-stimulated podocytes. Nrf2 knockdown partially blocked the protective effects of MitoQ on Ang II-induced mitochondrial fission and oxidative stress in podocytes. These results demonstrate that MitoQ exerts a protective effect in Ang II-induced mitochondrial injury in podocytes via the Keap1-Nrf2 signaling pathway.

## 1. Introduction

Podocytes are an important component of the glomerular filtration barrier, which is responsible for extremely complex filtration functions, and podocyte damage causes proteinuria and initiates the progression of chronic kidney disease (CKD) [1]. CKD is commonly accompanied by activation of the renin-angiotensin system (RAS) [2]. Angiotensin II (Ang II), a major component of the RAS, has been shown to directly induce podocyte injury [3, 4]. However, the precise mechanism of Ang II-induced podocyte injury remains elusive.

Accumulating evidence suggests a critical role for oxidative stress in podocyte injury [5, 6]. Interestingly, Ang II stimulates intracellular reactive oxygen species (ROS) formation and participates in the pathogenesis and development of CKD [7]. It has been demonstrated that ROS are mainly produced by the mitochondria [8], but the molecular mechanism by which Ang II stimulates mitochondria to produce a large amount of ROS is not completely clear [9]. Mitochondria undergo continuous fission and fusion to meet energy needs and to remove damaged mitochondria; collectively, these behaviors are referred to as mitochondrial dynamics [10]. Previous studies indicated that excessive mitochondrial fission could affect the structure and spatial organization of the respiratory chain and ATP synthase and impact electron transport and coupling, leading to the massive production of ROS and cell apoptosis [11]. We have recently reported that excessive mitochondrial fission and subsequent oxidative damage are critical components of podocyte injury [12]. Thus, it is reasonable to speculate that maintaining the homeostasis of mitochondrial dynamics may reduce Ang II-induced oxidative injury in podocytes.

Mitoquinone (MitoQ), a mitochondria-targeted antioxidant, is composed of coenzyme Q10 and TPP cations, and its affinity for mitochondria is hundredfold greater than that of traditional antioxidants [13]. Recent studies have suggested that MitoQ ameliorates oxidative stress in various diseases, including cardiovascular disease and hypoxia-induced pulmonary hypertension [14, 15]. In particular, MitoQ has been shown to protect against excessive mitochondrial fission and oxidative injury in tubular cells in the context of diabetic kidney disease (DKD) [16]. Therefore, we further hypothesized that MitoQ could protect against Ang II-induced podocyte injury by inhibiting mitochondrial fission.

Nuclear factor E2-related factor 2 (Nrf2) is a key transcription factor that regulates cellular antioxidant responses [17]. Kelch-like ECH-associated protein 1 (Keap1), a negative regulator of Nrf2, degrades Nrf2 through ubiquitination [18]. Emerging evidence has demonstrated that Nrf2 participates in the regulation of mitochondrial dynamics [19]. Intriguingly, a recent study showed that MitoQ attenuated mitochondrial fission and protected nucleus pulposus cells against oxidative injury by targeting the Keap1-Nrf2 pathway in intervertebral disc degeneration [20]. However, whether MitoQ prevents Ang II-induced podocyte injury and the underlying mechanism of such prevention remain unclear. In the present study, we investigated the protective effect of MitoQ on mitochondrial dynamics and oxidative injury in podocytes both *in vivo* and *in vitro* and evaluated the role of the Keap1-Nrf2 pathway in this process.

## 2. Materials and Methods

### 2.1. Animal Studies

Pathogen-free male C57BL/6 mice aged 8 weeks and weighing 18-22 g were used for animal experiments. The study was approved by the Committee on the Ethics of Animal Experiments of Renmin Hospital of Wuhan University. Following one week of adaptation, mice were randomly assigned to four groups: normal saline infusion group (an osmotic minipump (Alzet model 2004, CA) was embedded in mice, which received saline), saline infusion group receiving intraperitoneal injection of MitoQ (MCE, USA, 5 mg/kg, biweekly for 4 weeks) [16], Ang II infusion group (an osmotic minipump was embedded in mice, which received 700 ng/kg/min Ang II (Sigma-Aldrich, USA) infusion for 4 weeks), and Ang II infusion group receiving intraperitoneal injection of MitoQ. On the 28th day, after 24 h urine samples were collected for measurement of urinary proteins, the mice were euthanized, and kidney tissues were harvested for further studies.

### 2.2. Cell Culture and Treatments

Conditionally immortalized human podocytes were kindly gifted by Dr. Moin A. Saleem (Academic Renal Unit, Southmead Hospital, Bristol, UK). Podocytes were proliferated at 33°C in RPMI 1640 medium (HyClone, USA) supplemented with 10% fetal bovine serum (Gibco, USA), 100 *μ*g/mL streptomycin, 100 U/mL penicillin G (Thermo Fisher Scientific, USA), and 1× insulin-transferrin-selenium (ITS, Gibco). The cells were transferred to 37°C in ITS-free medium to induce differentiation. Differentiated podocytes were exposed to 10^−6^ M Ang II for 24 h to mimic the direct effect of Ang II on podocytes. Prior to Ang II exposure, the cells were pretreated with 50 nM or 100 nM MitoQ for 2 h. Nrf2 siRNA (TsingKe, China) was delivered to podocytes using HiPerFect (Qiagen, Germany) according to the manufacturer's instructions.

### 2.3. Immunofluorescence Staining

Paraffin-embedded kidney tissue sections were deparaffinized and subjected to antigen retrieval, which was performed under high pressure in citrate buffer (0.01 mol/L, pH 6.0) for 10 min. After blocking with 5% bovine serum albumin (BSA) for 1 h, the sections were incubated with primary antibodies overnight at 4°C (Nrf2, GeneTex, USA, #GTX103322; synaptopodin, Progen, Germany, #65194), followed by incubation with fluorochrome-conjugated secondary antibodies (Thermo Fisher Scientific) at 37°C for 2 h in the dark. Nuclei were counterstained with 4′,6′-diamidino-2-phenylindole (DAPI, Antgene, China). All images were taken with a fluorescence microscope (Olympus, Japan).

### 2.4. Immunohistochemical Staining

Paraffin-embedded kidney tissue sections were deparaffinized and subjected to antigen retrieval, which was performed under high pressure in citrate buffer (0.01 mol/L, pH 6.0) for 10 min, and then incubated with 3% hydrogen peroxide for 10 min. After blocking with 10% goat serum at room temperature for 30 min, the sections were incubated with anti-Nrf2 antibody (Nrf2, #GTX103322) overnight at 4°C and incubated with polymerized horseradish peroxidase-conjugated secondary antibody for 30 min. The sections were visualized with diaminobenzidine and counterstained with hematoxylin. Images were captured with a fluorescence microscope (Olympus).

### 2.5. Western Immunoblotting

Western immunoblotting was performed as described previously [3]. Briefly, total protein samples were separated by SDS-PAGE and then transferred to PVDF membranes (Sigma, USA), which were blocked with 5% nonfat milk for 1 h and incubated overnight at 4°C with the primary antibody (Nrf2, #GTX103322; Keap1, Novus, USA, #NBP1-83106; optic atrophy-1 (Opa1), ImmunoWay, USA, #YN2976; mitofusins2 (Mfn2), Abcam, USA, #ab124773; dynamin-related protein-1 (Drp1), Abcam, #ab56788; p-Drp1-Ser637, Cell Signaling Technology, USA, #4867; fission 1 (Fis1), GeneTex, #GTX111010; and GAPDH, Proteintech, China, #600004-1). HRP-labeled goat anti-rabbit/mouse IgG (H+L) antibody (Antgene, China) was used as the secondary antibody. The proteins were detected by a Bio-Rad imaging system.

### 2.6. MitoTracker Red Staining

MitoTracker Red (Invitrogen, USA) staining was performed as described previously [21]. Briefly, podocytes were incubated with 50 nM MitoTracker Red working solution for 30 min at 37°C and stained with DAPI to visualize the nuclei. All images were taken with a fluorescence microscope (Olympus).

### 2.7. Assessment of ROS, Mitochondrial Membrane Potential, and Adenosine Triphosphate (ATP) Content

Superoxide generation, mitochondrial membrane potential, and ATP content measurements were performed as previously described [22]. Briefly, ROS production in glomeruli and podocytes was evaluated by dihydroethidium (DHE, Invitrogen) and 2′,7′-dichlorodihydrofluorescein diacetate (H2-DCFDA, Beyotime, China) assays, respectively. Mitochondrial membrane potential was analyzed by JC-1 staining (Beyotime). ATP generation was measured with the ATP Determination Kit (Beyotime).

### 2.8. Apoptosis Assay

Podocyte apoptosis was assayed by flow cytometry using PE and 7-ADD double staining, and all procedures were performed according to the manufacturer's instructions (PE Annexin V Apoptosis Detection Kit, BioLegend, USA).

### 2.9. Statistical Analyses

GraphPad Prism 7 was used for statistical analysis. All values are expressed as the mean ± standard deviation (SD). Differences between all groups were determined by Student's *t*-test or one-way analysis of variance (ANOVA). *P* < 0.05 was considered statistically significant.

## 3. Results

### 3.1. MitoQ Ameliorated Glomerular and Podocyte Injury in Ang II-Infused Mice

**Figure 1:**
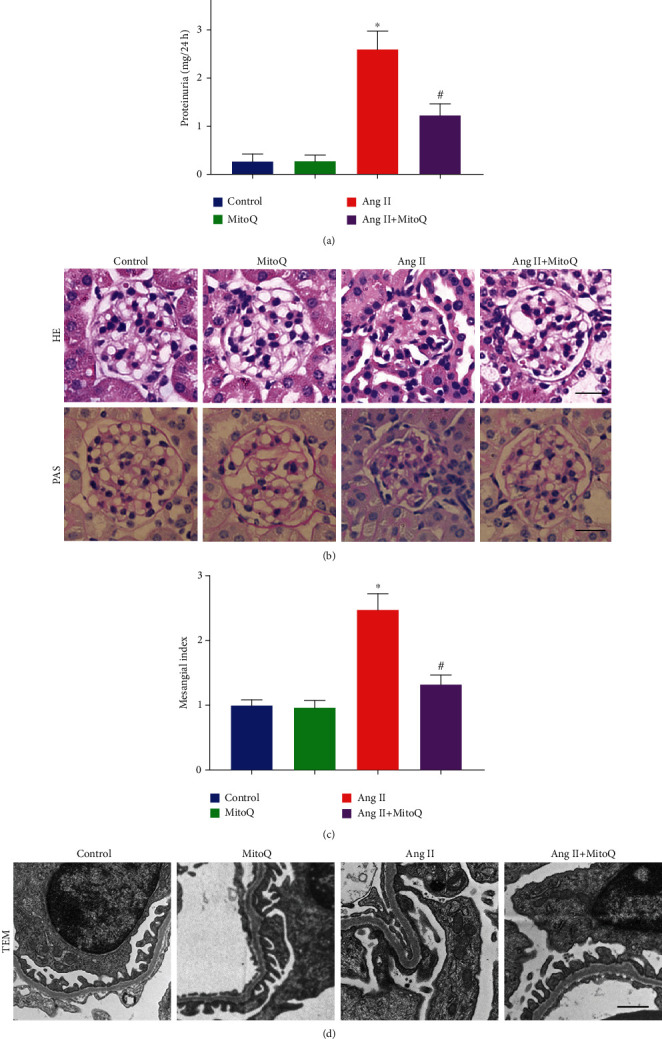
MitoQ prevents glomerular and podocyte injury in Ang II-infused mice. (a) 24 h urine total protein (UTP) levels in each group. (b) Representative images of kidney histology from each group (HE and PAS staining, original magnification, ×600). Scale bar = 20 *μ*m. (c) Quantitative analysis of the mesangial index from different experimental groups. (d) Representative glomerular ultrastructure from different groups by transmission electron microscopy (TEM, original magnification, ×10000), scale bar = 1 *μ*m. ^∗^*P* < 0.05 vs. the normal saline infusion group; ^#^*P* < 0.05 vs. the Ang II infusion group. *n* = 5.

An Ang II-infused mouse model was established to investigate whether MitoQ could prevent glomerular and podocyte injury. As shown in Figure 1(a), compared with saline-infused mice, Ang II-infused mice exhibited increased 24 h urine total protein (UTP) levels, and MitoQ treatment dramatically decreased UTP levels. Meanwhile, histological examination revealed notable morphological alterations in the glomeruli of Ang II-infused mice, manifested as mesangial matrix expansion and glomerulosclerosis. In addition, glomerular basement membrane (GBM) thickening and diffuse foot process fusion, a hallmark of podocyte injury, were observed by transmission electron microscopy (TEM) in Ang II-infused mice. Interestingly, these changes were significantly ameliorated by MitoQ administration (Figures 1(b)–1(d)).

### 3.2. MitoQ Promoted Podocyte Mitochondrial Fusion and Attenuated Oxidative Stress in Glomeruli from Ang II-Infused Mice

**Figure 2:**
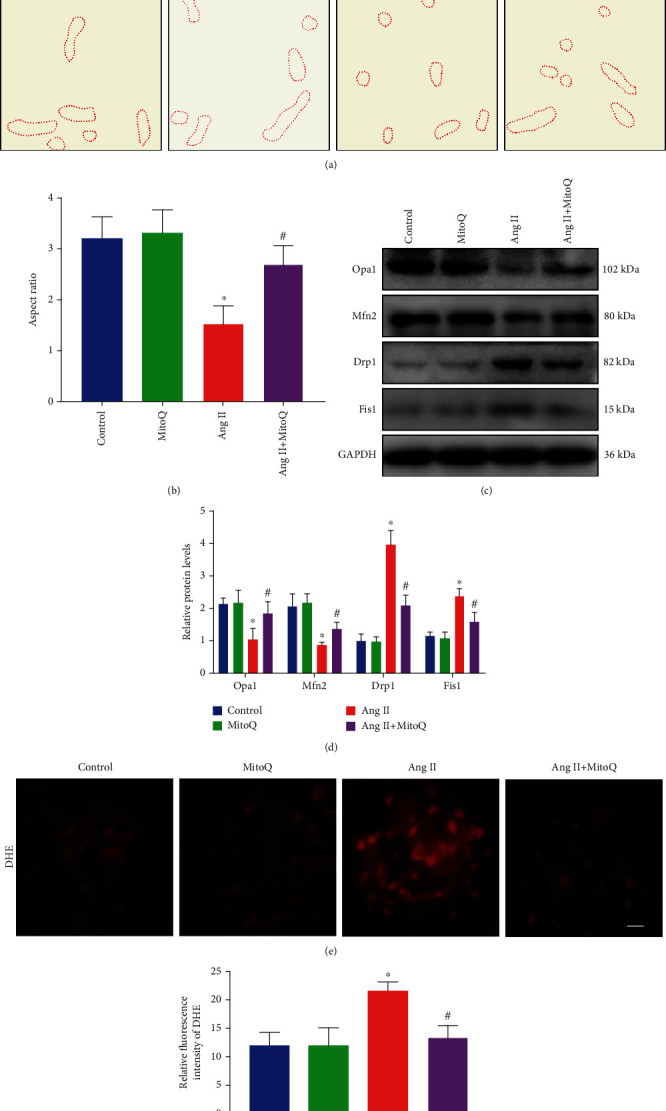
MitoQ ameliorates mitochondrial fission and ROS production in Ang II-infused mice. (a) Representative image of mitochondrial ultrastructure in podocytes from each group by TEM (original magnification, ×10000), scale bar = 1 *μ*m. (b) Quantitative analysis of the mitochondrial aspect ratio in the different groups. (c) Western blot analysis of Opa1, Mfn2, Drp1, and Fis1 in glomeruli from different groups. (d) Quantification of protein levels from (c). (e) DHE staining to detect oxidative stress in glomeruli from different groups (original magnification, ×600), scale bar = 20 *μ*m. (f) Quantification of fluorescence intensity from (e). ^∗^*P* < 0.05 vs. the normal saline infusion group; ^#^*P* < 0.05 vs. the Ang II infusion group. *n* = 5.

Emerging evidence has shown that podocyte mitochondrial fission leads to overproduction of ROS, which plays a critical role in the pathogenesis of CKD. Therefore, the present study tested the effects of MitoQ on mitochondrial injury *in vivo*. TEM was used to analyze the mitochondrial morphology and revealed increased mitochondrial fission in podocytes of the Ang II-infused mice compared with those in the saline-infused mice and reduced mitochondrial fragmentation in podocytes from mice administered MitoQ (Figures 2(a) and 2(b)). Moreover, decreased expression of mitochondrial fusion-related proteins (Opa1 and Mfn2) and increased expression of mitochondrial fission-related proteins (Drp1, p-Drp1, and Fis1) were detected in glomeruli from Ang II-infused mice. However, all these changes were rescued by MitoQ treatment (Figures 2(c) and 2(d) and Supplementary Fig S1a-b). Furthermore, DHE staining revealed increased ROS production in glomeruli from Ang II-infused mice, and MitoQ administration significantly alleviated ROS accumulation in the glomeruli of Ang II-infused mice (Figures 2(e) and 2(f)). Collectively, these results indicated that MitoQ treatment attenuated podocyte mitochondrial fragmentation and glomerular oxidative stress in Ang II-infused mice.

### 3.3. MitoQ Alleviated Ang II-Induced Podocyte Mitochondrial Fission *In Vitro*

**Figure 3:**
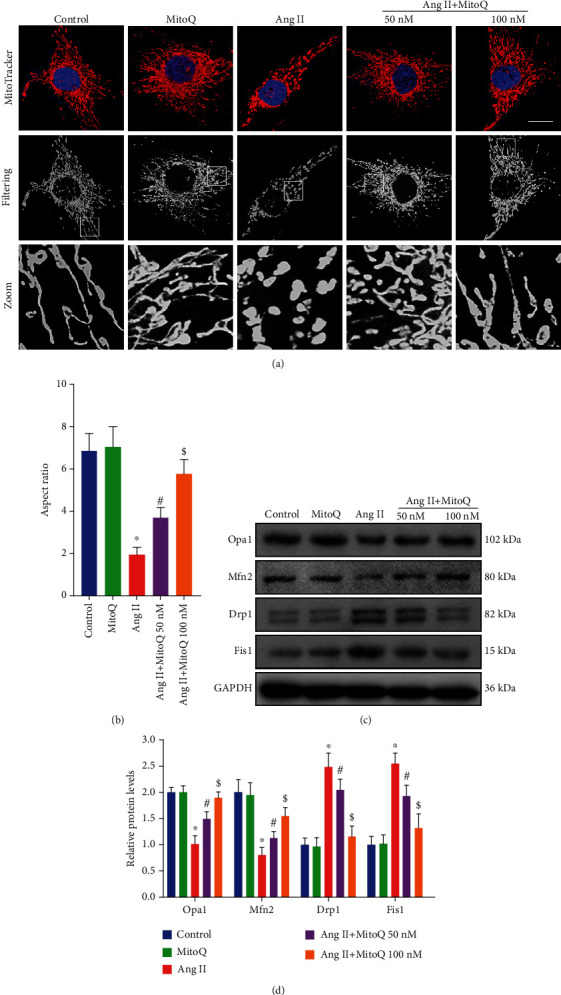
MitoQ alleviates mitochondrial fission *in vitro*. (a) Representative confocal microscopy image of MitoTracker Red staining in podocytes from each group (original magnification, ×1000), scale bar = 15 *μ*m, *n* = 5. (b) Quantitative analysis of the mitochondrial aspect ratio from (a). (c) Western blot analysis of Opa1, Mfn2, Drp1, and Fis1 in podocytes from different groups, *n* = 3. (d) Quantitative analysis of protein levels from (c). ^∗^*P* < 0.05 vs. control; ^#^*P* < 0.05 vs. Ang II; ^$^*P* < 0.05 vs. Ang II+MitoQ 50 nM.

Next, we investigated whether MitoQ could attenuate Ang II-induced podocyte mitochondrial fission *in vitro*. Mitochondria exhibited fragmented, punctuate, or round structures in Ang II-exposed podocytes, and MitoQ pretreatment significantly prevented the above-mentioned mitochondria morphology transformation in Ang II-exposed podocytes (Figures 3(a) and 3(b)). Meanwhile, the expression levels of Opa1, Mfn2, Drp1, p-Drp1, and Fis1 were detected by Western blotting. In line with the pattern of *in vivo* studies, MitoQ pretreatment partially corrected the overexpression of Drp1, p-Drp1, and Fis1 and the downregulation of Opa1 and Mfn2 in Ang II-exposed podocytes (Figures 3(c) and 3(d) and Supplementary Fig S1c-d).

### 3.4. MitoQ Attenuated Oxidative Stress, Mitochondrial Dysfunction, and Apoptosis in Ang II-Treated Podocytes

**Figure 4:**
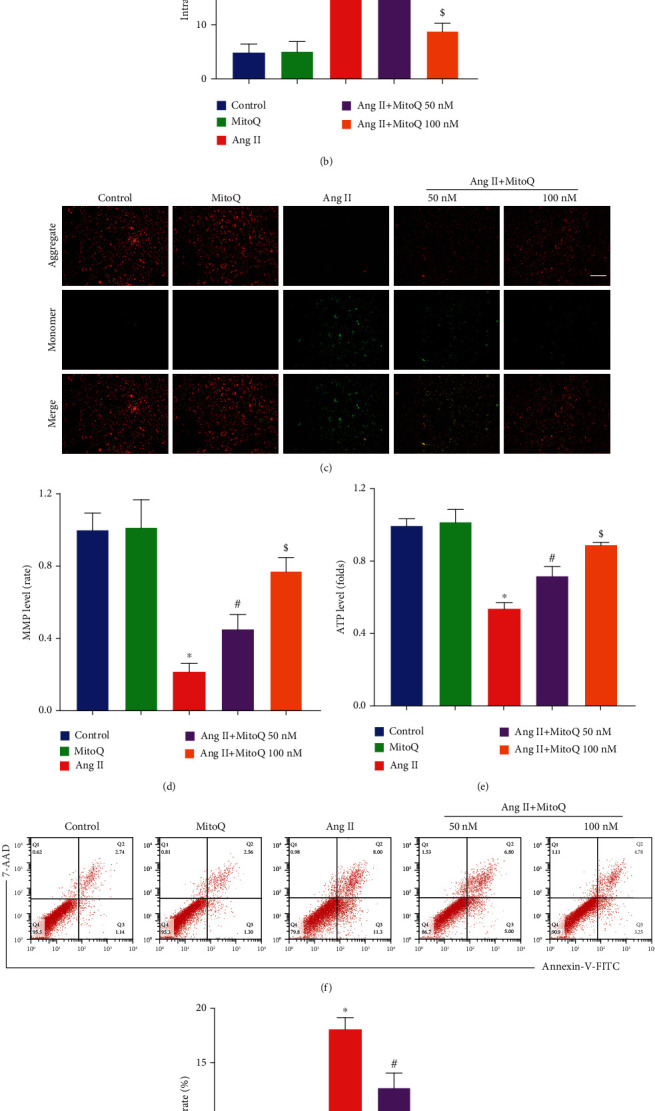
MitoQ attenuates oxidative stress, mitochondrial dysfunction, and apoptosis *in vitro*. (a) Representative DCFDA staining image of podocytes from each group (original magnification, ×100), scale bar = 100 *μ*m, *n* = 7. (b) Quantitative analysis of ROS content from (a). (c) JC-1 staining to assess membrane potential (original magnification, ×200), scale bar = 200 *μ*m, *n* = 4. (d) Quantitative analysis of membrane potential from (c). (e) Relative ATP content from each group, *n* = 3. (f) Podocyte apoptosis assessed by flow cytometry in different groups, *n* = 3. (g) The apoptosis rates in different groups from (f). ^∗^*P* < 0.05 vs. control; ^#^*P* < 0.05 vs. Ang II; ^$^*P* < 0.05 vs. Ang II+MitoQ 50 nM.

MitoQ has been proven to ameliorate mitochondrial oxidative injury through the inhibition of mitochondrial fission in nucleus pulposus cells and renal tubular cells [16, 20], but its protective effect on podocytes is unclear. ROS production in podocytes was detected by DCFDA staining. We found that Ang II exposure markedly increased ROS generation and MitoQ pretreatment significantly attenuated the elevated ROS production (Figures 4(a) and 4(b)). To further investigate the effects of MitoQ on mitochondrial function, the mitochondrial membrane potential (MMP) and cellular ATP generation in podocytes were evaluated. Ang II exposure dramatically decreased MMP and cellular ATP content, and these alterations were notably prevented by MitoQ pretreatment (Figures 4(c)–4(e)). To explore whether MitoQ is able to improve the outcome of podocyte injury induced by Ang II, podocyte apoptosis was assayed by flow cytometry, and we observed that MitoQ pretreatment significantly reduced apoptotic podocytes induced by Ang II (Figures 4(f) and 4(g)). These results indicate that MitoQ attenuates oxidative stress and mitochondrial dysfunction through the inhibition of mitochondrial fission. These results suggest that inhibition of mitochondrial fission is a key process by which MitoQ attenuates oxidative stress and mitochondrial dysfunction in podocytes.

### 3.5. Effects of MitoQ on the Expression of the Mitochondrial Oxidative Stress-Associated Protein Keap1 and Nrf2

**Figure 5:**
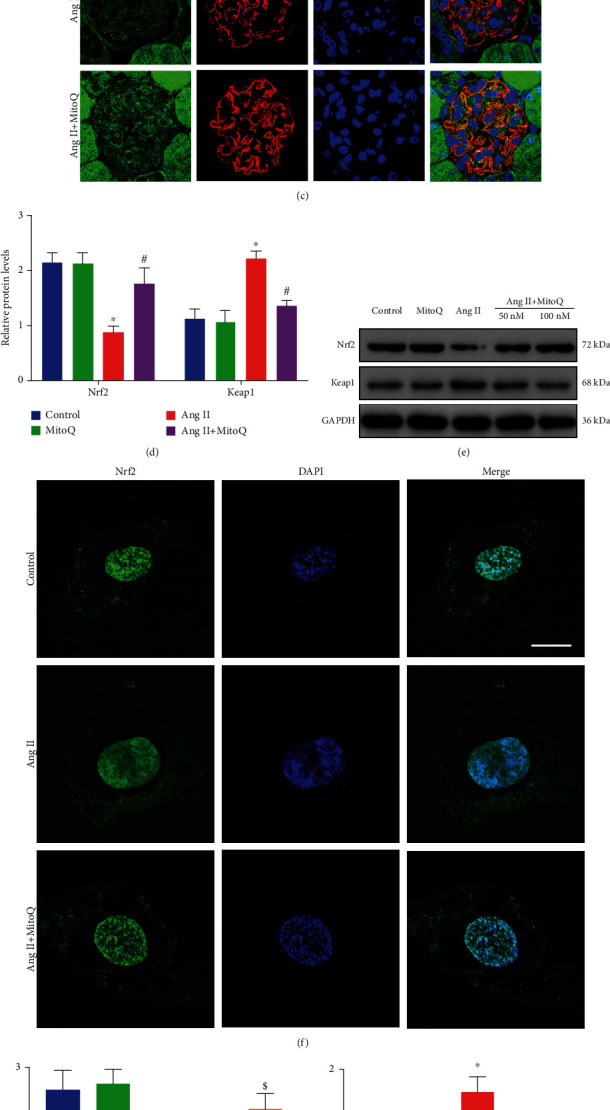
Effects of MitoQ on the expression of Keap1 and Nrf2 in podocytes *in vivo* and *in vitro*. (a) Western blot analysis of Keap1 and Nrf2 in glomeruli from different groups, *n* = 3. (b) Representative immunohistochemistry staining of Nrf2 in glomeruli from different groups (original magnification, ×600), scale bar = 20 *μ*m. (c) Representative fluorescence staining of Nrf2 (green), synaptopodin (podocytes, red), and DAPI (blue) in podocytes from different groups (original magnification, ×1000), scale bar = 10 *μ*m, *n* = 5. (d) Quantitative analysis of protein levels from (a). (e) Western blot analysis of Keap1 and Nrf2 in podocytes *in vitro*, *n* = 3. (f) Representative fluorescence staining of Nrf2 in podocytes *in vitro* (original magnification, ×1000), scale bar = 15 *μ*m, *n* = 5. (g, h) Quantitative analysis of protein levels from (e). (i) Quantitative analysis of the nuclear translocation of Nrf2. ^∗^*P* < 0.05 vs. control; ^#^*P* < 0.05 vs. Ang II; ^$^*P* < 0.05 vs. Ang II+MitoQ 50 nM.

The above results indicated that MitoQ notably alleviated Ang II-induced mitochondrial fragmentation and oxidative stress in podocytes both *in vivo* and *in vitro*. However, the underlying molecular mechanism by which MitoQ protects against mitochondrial injury remains unidentified. Since the Keap1/Nrf2 signaling pathway plays a key role in antioxidative stress, we then investigated whether Nrf2 activation was involved in the protective effects of MitoQ on mitochondrial injury in podocytes. Western blotting results showed increased Keap1 and decreased Nrf2 expression in glomeruli from Ang II-infused mice compared with saline-infused mice (Figures 5(a) and 5(d)). Moreover, decreased Nrf2 expression in glomeruli and podocytes from Ang II-infused mice was also confirmed by immunohistochemical and immunofluorescence double staining, respectively. Meaningfully, MitoQ partially rescued the abnormal expression patterns of these proteins (Figures 5(b) and 5(c)). Consistent with the results of *in vivo* studies, increased Keap1 and decreased Nrf2 expression were also shown in Ang II-stimulated podocytes *in vitro* and restored by MitoQ pretreatment (Figures 5(e), 5(g), and 5(h)). In addition, the nuclear translocation of Nrf2 was monitored by immunofluorescence, and the nuclear location of Nrf2 expression was significantly decreased in Ang II-stimulated podocytes. MitoQ pretreatment dramatically reversed the nuclear translocation of Nrf2 induced by Ang II (Figures 5(f) and 5(i)). Taken together, these data suggest that Nrf2 activation may be involved in the protective effect of MitoQ in podocytes.

### 3.6. Silencing of Nrf2 Abolished the Protective Effect of MitoQ on Ang II-Induced Mitochondrial Fission in Podocytes

**Figure 6:**
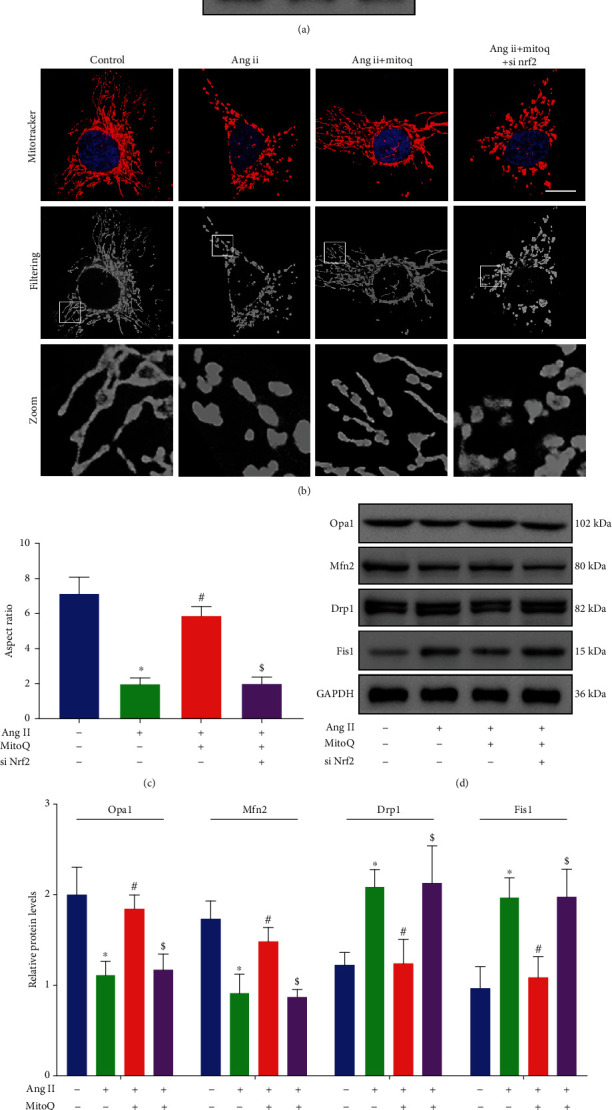
Nrf2 knockdown affects the protective effect of MitoQ on Ang II-induced mitochondrial fission. (a) Western blot analysis of Nrf2 in podocytes transfected with Nrf2 siRNA or siRNA scrambles, *n* = 3. (b) Representative confocal microscopy image of MitoTracker Red staining in podocytes from each group (original magnification, ×1000), scale bar = 15 *μ*m, *n* = 4. (c) Quantitative analysis of the mitochondrial aspect ratio from (b). (d) Western blot analysis of Opa1, Mfn2, Drp1, and Fis1 in podocytes from different groups, *n* = 3. (e) Quantitative analysis of protein levels from (d). ^∗^*P* < 0.05 vs. control; ^#^*P* < 0.05 vs. Ang II; ^$^*P* < 0.05 vs. Ang II+MitoQ 100 nM.

To examine how Nrf2 contributes to the protective effects of MitoQ on mitochondrial dynamics, Nrf2 siRNA was used to knockdown the expression of Nrf2 *in vitro* (Figure 6(a)). MitoTracker Red staining revealed that the protective effect of MitoQ on mitochondrial morphology was blocked by Nrf2 knockdown (Figures 6(b) and 6(c)). In accordance with these findings, Western blotting results demonstrated that the increased expression of Drp1, p-Drp1, and Fis1 and the decreased expression of Opa1 and Mfn2 in Ang II-stimulated podocytes were rescued by MitoQ and that this effect was partially abolished by Nrf2 siRNA transfection (Figures 6(d) and 6(e) and Supplementary Fig S1e-f). These results suggest that Nrf2 signaling is involved in the protection of MitoQ against Ang II-induced mitochondrial fission in podocytes.

### 3.7. MitoQ Attenuated Oxidative Stress, Mitochondrial Dysfunction, and Podocyte Apoptosis Partially via Nrf2 Signaling

**Figure 7:**
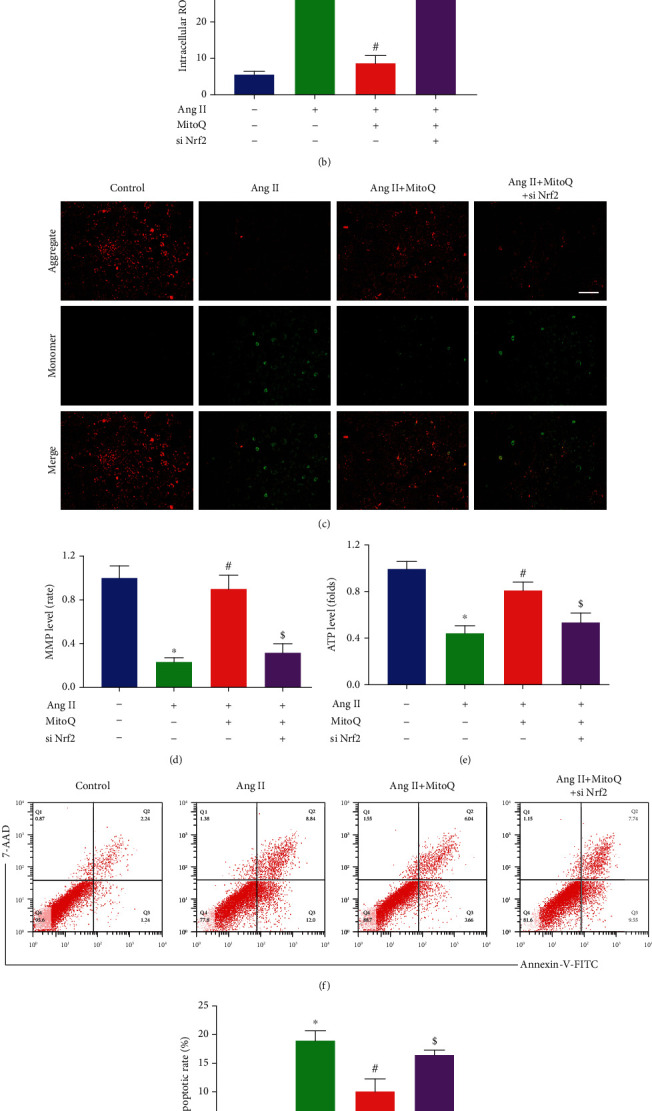
MitoQ attenuated oxidative stress, mitochondrial dysfunction, and podocyte apoptosis partially via Nrf2 signaling. (a) Representative DCFDA staining image of podocytes from each group (original magnification, ×100), scale bar = 200 *μ*m, *n* = 4. (b) Quantitative analysis of ROS content from (a). (c) Representative JC-1 staining image of podocytes from each group (original magnification, ×200), scale bar = 100 *μ*m, *n* = 4. (d) Quantitative analysis of membrane potential from (c). (e) Relative ATP content in podocytes from each group, *n* = 3. (f) Podocyte apoptosis assessed by flow cytometry in different groups, *n* = 3. (g) The apoptosis rates in different groups from (f). ^∗^*P* < 0.05 vs. control; ^#^*P* < 0.05 vs. Ang II; ^$^*P* < 0.05 vs. Ang II+MitoQ 100 nM.

We further investigated whether Nrf2 knockdown could attenuate the protective effects of MitoQ on Ang II-induced oxidative damage in podocytes. DCFDA staining showed that MitoQ reduced ROS accumulation in Ang II-stimulated podocytes, and Nrf2 siRNA transfection abolished this effect (Figures 7(a) and 7(b)). Similarly, Ang II-induced mitochondrial dysfunction was reversed by MitoQ and was partially eliminated by Nrf2 siRNA transfection, as evidenced by JC-1 staining and ATP content assays (Figures 7(c)–7(e)). Furthermore, knockdown of Nrf2 significantly but incompletely abolished the protective effects of MitoQ on Ang II-induced podocyte apoptosis (Figures 7(f) and 7(g)). These results suggest that the protective effects of MitoQ on podocytes are in part dependent on Nrf2.

## 4. Discussion

The present study demonstrated that treatment with MitoQ could attenuate podocyte injury and glomerulosclerosis in Ang II-infused mice. MitoQ is able to maintain mitochondrial dynamics homeostasis and reduce Ang II-induced mitochondrial fission and subsequent oxidative stress in podocytes. Moreover, the possible mechanism of these effects was further explored. These results suggest that the protective effects of MitoQ on podocytes are mediated in part by Nrf2 and that Nrf2 may affect mitochondrial dynamics by regulating the expression of Opa1, Mfn2, Drp1, p-Drp1, and Fis1.

Mitochondrial fusion and fission dynamics play a critical role in the pathogenesis and development of kidney disease [23–27]. Mitochondrial fission is mainly regulated by Drp1 and its receptors, including Fis1, Mff (mitochondrial fission factor), and MiD49/MiD51 (mitochondrial dynamics proteins of 49 and 51 kDa) [5, 28]. During the fission process, Drp1 is recruited from the cytosol to the mitochondrial outer membrane by its receptors and forms a ring-like oligomer that mediates the partition of mitochondria [29]. In addition, Drp1 phosphorylation at Ser637 residue contributes to the mitochondrial fission process [30]. Conversely, Mfn1/2, two dynamin-related GTPases, mediate mitochondrial fusion by tethering mitochondrial outer membranes [23]. Opa1, another dynamin-related GTPase, facilitates inner membrane fusion and the stabilization of cristae [5]. It has been shown that Opal degradation is involved in the repression of mitochondrial fusion [31, 32]. Dysregulation of the balance between the fusion and fission proteins alters mitochondrial morphology and causes mitochondrial dysfunction [29, 33, 34]. Our and others' studies demonstrated excessive mitochondrial fission in podocytes in DKD patients and DKD animal models; such excessive mitochondrial fission could lead to ROS overproduction and mitochondrial dysfunction [12, 30, 35, 36]. Overactivation of the RAS is the main risk factor for CKD and DKD and is closely related to kidney oxidative stress [37–40], but its role in mitochondrial dynamics and oxidative stress in podocytes has rarely been studied. This study is the first to investigate the correlation between Ang II, a representative component of the RAS, and podocyte mitochondrial dynamics. The findings here show that Ang II stimulation upregulated the expression of Drp1, p-Drp1, and Fis1 and downregulated the expression of Mfn2 and Opa1, resulting in mitochondrial fragmentation and oxidative injury in podocytes.

Although accumulated evidence suggests that mitochondrial fission and oxidative stress participate in CKD progression [41, 42], there have been only limited studies on whether reducing mitochondrial fission and ROS production in podocytes by pharmacological approaches could reduce kidney damage. The application of angiotensin-converting enzyme inhibitors (ACEIs) or angiotensin-receptor blockers (ARBs) in CKD patients leads to partial relief of oxidative stress in the kidney [7], and the effects of antioxidant interventions targeting the level of total cellular redox status are disappointing, since traditional antioxidants are not well taken up by mitochondria. The mitochondria-targeted antioxidant MitoQ can quickly enter cells and be absorbed by mitochondria [43]. To the best of our knowledge, no studies have focused on the effect of MitoQ on podocyte injury induced by Ang II. Podocytes and their foot processes play a crucial role in establishing the selective permeability of the glomerular filtration barrier, and podocyte injury is associated with proteinuria and glomerulosclerosis [1]. This study demonstrated that MitoQ significantly alleviated proteinuria, foot process fusion, and glomerulosclerosis. In neurodegeneration, intervertebral disc degeneration, and DKD animal models, MitoQ exhibited a protective effect by maintaining the homeostasis of mitochondrial dynamics [16, 20, 44]. Thus, we further investigated the protective effects of MitoQ on podocytes and whether this effect was due to the regulation of mitochondrial dynamics. In line with those studies, we have shown that MitoQ significantly attenuated mitochondrial fission by downregulating the expression of Drp1, p-Drp1, and Fis1 and upregulating the expression of Mfn2 and Opa1.

The present study also explored the underlying mechanism by which MitoQ regulated mitochondrial dynamics in Ang II-stimulated podocytes. Nrf2 is a crucial transcription factor that regulates the production of antioxidants to maintain cellular redox balance [17]. As a negative regulator of Nrf2, Keap1 interacts with Nrf2 to cause Nrf2 polyubiquitination and subsequent degradation [18]. Sabouny et al. [45] recently demonstrated that stress-activated Nrf2 could increase proteasomal activity then promote the degradation of Drp1, which lead to decreased mitochondrial fission. In addition to regulating mitochondrial dynamics by affecting mitochondrial fission/fusion proteins, Nrf2 may also indirectly regulate mitochondrial dynamics by affecting mitochondrial biosynthesis and mitophagy. Dinkova et al. [46] suggested that Nrf2 could maintain the level of PGC1*α* and increase mitochondrial biogenesis to replace fragmented mitochondria in the context of stress conditions. Moreover, activated Nrf2 can promote mitophagy via the PINK/Parkin pathway to remove fragmented mitochondria [47]. It has been demonstrated that activation of Nrf2 or inhibition of Keap1 protects against kidney diseases such as acute kidney disease, autosomal dominant polycystic kidney disease, and CKD [48–50]. Zhou et al. [51] reported that activation of the Nrf2 antioxidant response could alleviate podocytopathy, glomerular injury, and proteinuria induced by doxorubicin. Moreover, Nrf2 contributed to the restoration of mitochondrial dynamics in tubular cells treated with MitoQ in DKD [16]. Therefore, it is reasonable to speculate that MitoQ is able to regulate podocyte mitochondrial dynamics through Nrf2.

To verify this hypothesis, we used different methods to detect the expression of Keap1 and Nrf2 in podocytes and revealed that Keap1 expression was increased and Nrf2 expression was decreased in Ang II-infused mice. Moreover, the reduction in Nrf2 levels and nuclear translocation was accompanied by significant mitochondrial fragmentation and oxidative stress in podocytes. However, these alterations were significantly rescued by MitoQ administration, and knockdown of Nrf2 in podocytes by using siRNA abolished the protective effects of MitoQ on mitochondrial fission and oxidative stress induced by Ang II, providing further support for the hypothesis that Nrf2 may play a crucial role in the regulation of mitochondrial dynamics in podocytes. However, activation of Nrf2 serves as a double-edged sword for oxidative stress [52]. Insufficiency of Nrf2 leads to disruption of redox balance, resulting in accumulation of ROS and free radicals. In contrast, continuous upregulation of Nrf2 could increase the level of antioxidants such as glutathione (GSH) and thioredoxin (TXN), resulting in blunt sensitivity of ROS-dependent inflammation and apoptosis [52, 53]. Furthermore, the functional role of Nrf2 in kidney disease is controversial; our and many other studies suggested that Nrf2 exert an antioxidant effect and delay the progression of renal damage [54–56]. However, Rush et al. [56] demonstrated that sustained activation of the Nrf2 pathway could induce podocyte injury and increase proteinuria in a CKD animal model. Thus, all these studies indicated that it may be necessary to monitor the activity level of Nrf2 in CKD patients.

In the present study, the exact mechanism by which MitoQ regulates Nrf2 and Keap1 was not studied. In addition, the translocation of Nrf2 into the nucleus promotes the transcription of antioxidative stress factors such as NAD(P)H quinone dehydrogenase 1 (NQO-1) and heme oxygenase 1 (HO-1) [57]. Although this study demonstrated that MitoQ promoted Nrf2 nuclear translocation, the expression levels of NQO-1 and HO-1 were not evaluated.

In conclusion, this study demonstrates for the first time that MitoQ has a role in protecting against podocyte injury induced by Ang II. The mechanism underlying its effects may be attributed to the maintenance of mitochondrial dynamics via Nrf2, which results in decreased mitochondrial fission and oxidative injury. These findings strongly support the therapeutic value of MitoQ in RAS-associated podocyte injury.

## Data Availability

The data that support the findings of this study are available from the corresponding author upon reasonable request.

## Supplementary Materials

Supplementary Materials Supplementary Fig S1: (a) Western blot analysis of pDrp1(637) in glomeruli from different groups. (b) Quantification of protein levels from panel a; ^∗^*P* < 0.05 vs. normal saline infusion group; ^#^*P* < 0.05 vs. Ang II infusion group. *n* = 5. (c) Western blot analysis of pDrp1(637) in podocytes from different groups. (d) Quantification of protein levels from panel c; ^∗^*P* < 0.05 vs. control; ^#^*P* < 0.05 vs. Ang II; ^$^*P* < 0.05 vs. Ang II+MitoQ 50 nM, *n* = 3. (e) Western blot analysis of pDrp1(637) in podocytes from different groups. (f) Quantification of protein levels from panel e; ^∗^*P* < 0.05 vs. control; ^#^*P* < 0.05 vs. Ang II; ^$^*P* < 0.05 vs. Ang II+MitoQ 100 nM, *n* = 3.
