# Supplementary material for: Mitoquinone Protects Podocytes from Angiotensin II-Induced Mitochondrial Dysfunction and Injury via the Keap1-Nrf2 Signaling Pathway
Source: Oxid Med Cell Longev. 2021 Aug 13;2021:1394486. doi: 10.1155/2021/1394486 (PMC8380182; doi:10.1155/2021/1394486)
Supplement: Supplementary Materials — Supplementary Fig S1: (a) Western blot analysis of pDrp1(637) in glomeruli from different groups. (b) Quantification of protein levels from panel a; ∗P < 0.05 vs. normal saline infusion group; #P < 0.05 vs. Ang II infusion group. n = 5. (c) Western blot analysis of pDrp1(637) in podocytes from different groups. (d) Quantification of protein levels from panel c; ∗P < 0.05 vs. control; #P < 0.05 vs. Ang II; $P < 0.05 vs. Ang II+MitoQ 50 nM, n = 3. (e) Western blot analysis of pDrp1(637) in podocytes from different groups. (f) Quantification of protein levels from panel e; ∗P < 0.05 vs. control; #P < 0.05 vs. Ang II; $P < 0.05 vs. Ang II+MitoQ 100 nM, n = 3. [file 1394486.f1.docx]

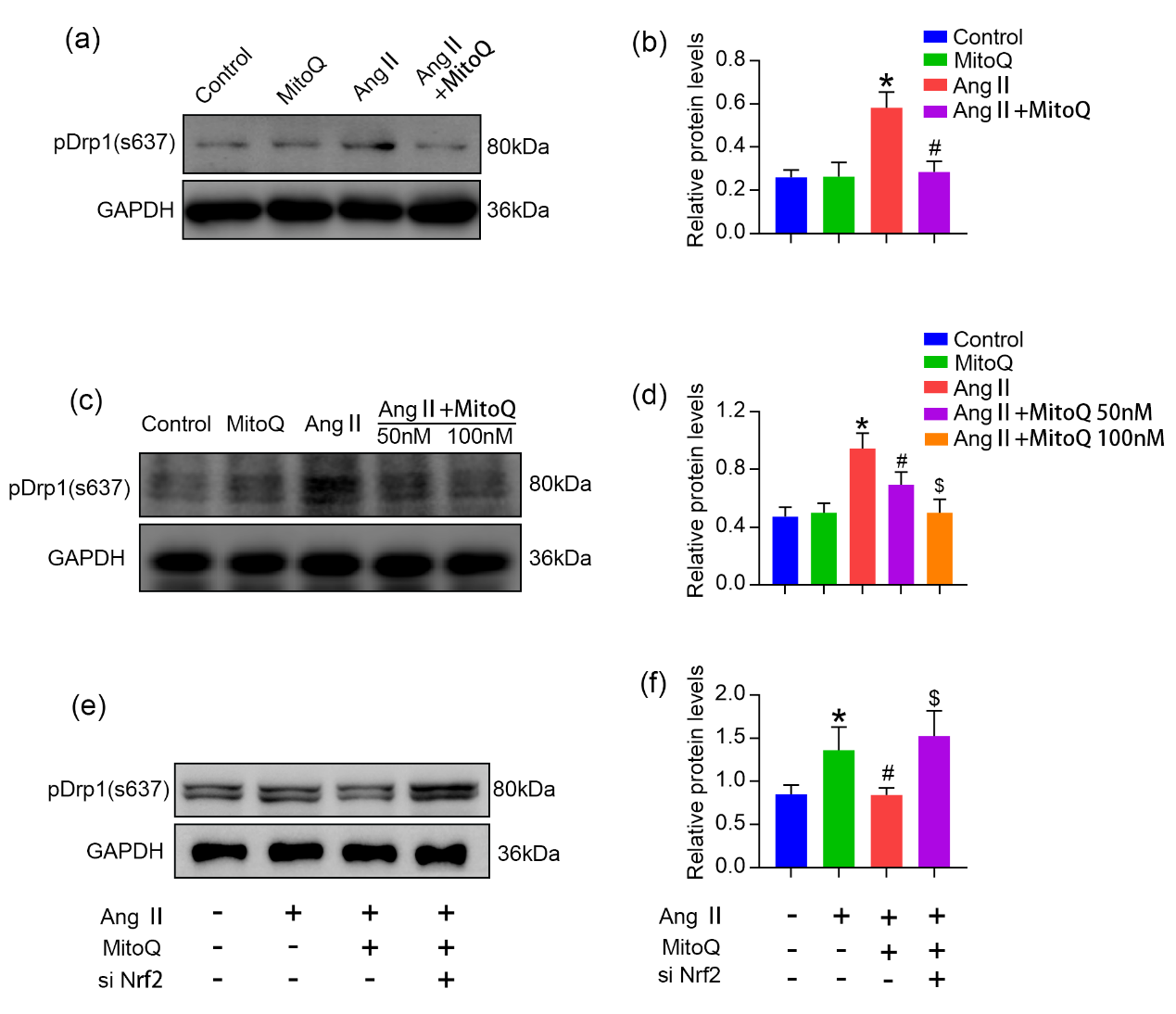


Supplementary Fig S1: (a): Western blot analysis of pDrp1(637) in glomeruli from different groups. (b): Quantification of protein levels from panel a, *P < 0.05 vs normal saline infusion group; #P < 0.05 vs Ang II infusion group. n=5. (c): Western blot analysis of pDrp1(637) in podocytes from different groups. (d): Quantification of protein levels from panel c, *P < 0.05 vs control; #P < 0.05 vs Ang II, $P < 0.05 vs Ang II+MitoQ 50nM, n=3. (e): Western blot analysis of pDrp1(637) in podocytes from different groups. (f): Quantification of protein levels from panel e, *P < 0.05 vs control; #P < 0.05 vs Ang II, $P < 0.05 vs Ang II+MitoQ 100nM, n=3.
